# A New Gilliam Genotypic Variant of *Orientia tsutsugamushi* in Human Scrub Typhus Cases from South India

**DOI:** 10.3390/microorganisms13122670

**Published:** 2025-11-24

**Authors:** Steny Vallomkottu Joseph, Krishnamoorthy Nallan, Gopinathan Rajan, Amudhan Murugesan, Renu Govindarajan, Raju Sivadoss, Ramkumar Ramalingam, Rajarathinam Kannan Madhumitha, Sucila Thangam Ganesan, Suria Kumar Jayakumar, Manju Rahi, Paramasivan Rajaiah

**Affiliations:** 1 Unit of Molecular Biology and Diagnosis, ICMR-Vector Control Research Centre, Field Station, No. 4, Sarojini Street, Madurai 625002, India; stenyjosephv@gmail.com (S.V.J.);; 2 Department of Microbiology, Government Medical College and Hospital, Dindigul 624003, India; 3 Department of Microbiology, Government Medical College and Hospital, Theni 625531, India; 4 Directorate of Public Health & Preventive Medicine, Chennai 600006, India; 5 Director, ICMR-Vector Control Research Centre, Puducherry 605006, India

**Keywords:** scrub typhus, *O. tsutsugamushi*, genetic diversity, Gilliam, genotype, Karp, Kato, Ot-TJTN

## Abstract

Scrub typhus, caused by *Orientia tsutsugamushi* (Ot), is a re-emerging public health concern across Southeast Asia. Although multiple Ot strains have been identified in endemic regions, their genetic characterization in India remains limited. We analyzed Ot strains from humans by targeting the *GroEL* and 56-kDa TSA genes. A total of 105 serum samples were subjected to PCR amplification and phylogenetic analysis for the *GroEL* gene, of which 33 (31.4%) were positive. Phylogenetic reconstruction revealed four major clades: Karp, Kato, Ot-TJTN (novel Ot-Thanjavur-Tamil Nadu), and the Gilliam group. Among the 33 PCR positives, 11 sequences clustered into a distinct monophyletic clade within the Gilliam group but diverged significantly from known classical Gilliam strains. The overall mean nucleotide diversity (π) was 0.02 (2%), while the divergence between these 11 sequences and the Gilliam strain was 0.039 (3.9%). The observed divergence indicates that these sequences represent the first identified Indian Gilliam variant (IG-v), showing marked genetic distinction from classical Gilliam and other related strains. Further analysis of the *56-kDa* gene from the 11 IG-v samples revealed phylogenetic incongruence between the *GroEL* and *56-kDa* genes, indicating antigenic reassortment involving three clades: Karp-like (n = 7), Ot-TJTN-like (n = 3), and Gilliam (n = 1). Similarity plot and recombination analyses, using *56-kDa* Ot-TJTN and Karp-like clades as queries, against Ot reference strains revealed preliminary evidence of genetic exchange. These findings highlight the possible role of recombination and antigenic shift in driving the evolutionary dynamics and genetic diversity of Ot in this region. Notably, the identification of an IG-v marks a significant advancement in our understanding of the circulating Ot strains. This finding holds important implications for refining molecular diagnostics, enhancing serological assays, and developing broadly protective vaccines targeting region-specific variants.

## 1. Introduction

Scrub typhus, once a neglected febrile illness, has re-emerged as a significant public health threat across Asia and beyond [1]. It is caused by an obligate intracellular bacterium, *O. tsutsugamushi,* and is transmitted by the larval stage (chiggers) of trombiculid mites, especially *Leptotrombidium deliense*, which acts as both vector and reservoir [2]. Globally, 2 billion people are at risk, and an estimated 1 million cases occur annually [1]. Underreporting of scrub typhus is common, as the characteristic clinical feature, an eschar at the bite, is frequently absent or unnoticed. The absence of this feature and fever symptoms that overlap with other febrile illnesses often hampers the timely and accurate diagnosis of scrub typhus. Moreover, insensitive diagnostic methods often delay identification [3].

Mite vectors adapt to local environmental conditions, which play a key role in the regional distribution of *O. tsutsugamushi*. Within these mite populations, genetic recombination can produce new strains, increasing the pathogen’s antigenic variability [4,5]. This is further enhanced by the bacterium’s genome, which contains many repetitive sequences. These features promote intragenomic rearrangements and lateral gene transfer [4] and result in the emergence of many unique, region-specific genotypes, thus complicating efforts for disease surveillance and control [6]. This extensive diversity poses significant challenges to serodiagnosis and vaccine development, as immunity is often strain-specific and reinfections with different strains are common [7].

In India, scrub typhus is endemic, with a high incidence in the rural and semi-urban regions. However, molecular data on circulating *O. tsutsugamushi* remain regionally limited [3]. While over 40 distinct genotypes have been reported globally [8], genotypic information from India is largely restricted to certain northern, northeastern, and southern regions [9]. A cohort study in South India reported that approximately 1 in 20 participants were affected by *O. tsutsugamushi* infection [10]. Recent clinical cases have identified *O. tsutsugamushi* as an emerging cause of acute encephalitis syndrome (AES), previously attributed primarily to viral infections such as Japanese encephalitis [11]. Further investigations have revealed a wide range of circulating genotypes with varying pathogenicity and strain-dependent mortality rates of up to 50%. These genotypic differences, along with variable antibiotic susceptibilities, pose significant diagnostic challenges [12]. A recent molecular study, based on clinical samples from Thanjavur Medical College Hospital in Tamil Nadu, identified a novel genotype designated as Ot-TJTN (Ot-Thanjavur-Tamil Nadu) [13]. This newly detected variant signifies the unrecognized strain of *O. tsutsugamushi* in southern India and emphasizes the need for continued molecular surveillance to monitor emerging genotypes and regional strain evolution.

Nevertheless, while type-specific antigen genes such as *56-kDa*, *47-kDa*, surface cell antigen (SCA), and 22-kDa are used for genotyping *O. tsutsugamushi*, the *56-kDa* gene remains the most commonly used marker due to its antigenic variability. This immunodominant nature makes it susceptible to host-driven selective pressures, which can obscure true evolutionary relationships [14]. Hence, its use as a phylogenetic marker has limitations [15]. The *56-kDa* gene's variable domain I-III (VDI-III) represents higher hypervariability levels than the entire gene [16]. In addition, numerous mobile genetic elements in the *O. tsutsugamushi* genome facilitate lateral gene transfer, contributing to genetic diversity. In contrast, the *GroEL* gene, which encodes the 60-kDa heat shock protein (HSP), shows conserved but informative polymorphisms, making it a more reliable marker for phylogenetic analysis and strain differentiation [15]. A systematic analysis of Gilliam-related strains has not yet been conducted in this region. To gain a more comprehensive understanding of the genetic nature of *O. tsutsugamushi*, both markers were examined. This study was carried out retrospectively on ELISA-processed clinical samples collected from human cases attending government hospitals in the Theni and Dindigul districts of Tamil Nadu. These regions, located at the foothills of the Western Ghats, are ecologically diverse and have reported cases of scrub typhus fever throughout the year [17,18,19]. However, no information on circulating *O. tsutsugamushi* genotypes is available, except for a recent study from the Theni district by Nallan et al. (2023) [20], making these areas important for studying the molecular characteristics of circulating *O. tsutsugamushi* strains.

## 2. Materials and Methods

### 2.1. Study Area

The Theni and Dindigul districts are situated along the foothills of the Western Ghats in southwestern Tamil Nadu (9°45′–10°30′ N, 77°15′–78°00′ E) and are known endemic zones for scrub typhus [17,19]. These regions are characterized by a tropical climate with seasonal monsoons, moderate to high humidity, and a diverse landscape with forest fringes and agricultural fields. These environmental factors favor chigger infestations in small mammal hosts across both rural and urban areas of these districts (unpublished). Consequently, deaths and scrub typhus cases have been routinely reported by state health authorities in recent times [17,18,19].

### 2.2. Sample Collection

Blood samples were collected from patients with acute febrile illness attending government public hospitals in the Theni and Dindigul districts, Tamil Nadu. Patients suspected of having scrub typhus were screened using a commercial IgM ELISA kit (Scrub Typhus Detect™ IgM ELISA, InBios International, Inc., Seattle, WA, USA). Only those who tested positive for scrub typhus in the ELISA were retrospectively selected for molecular analysis, resulting in 105 ELISA-positive samples. Blood samples were collected and transported under cold-chain conditions to the ICMR-Vector Control Research Centre (VCRC), Field Station, Madurai. The samples were stored at −20 °C until processing. Genomic DNA was extracted and analyzed using nested PCR assays targeting the *GroEL* and *56-kDa TSA* genes of *O. tsutsugamushi* for genotypic characterization. Ethical approval for this study was obtained from the Institutional Human Ethics Committee of ICMR-VCRC (approval no: TMC/IHEC: 1118/2023; 27 July 2023).

### 2.3. Molecular Detection and Genotyping of O. tsutsugamushi

#### 2.3.1. Genomic DNA Extraction and *GroEL* Gene Amplification

Genomic DNA was extracted from ELISA-positive human serum samples using the QIAamp DNA Blood Mini Kit (Qiagen, Hilden, Germany; Cat. No. 51104), following the manufacturer’s instructions. The DNA concentration and purity were assessed, and aliquots were stored at −20 °C until further analysis. Nested PCR targeting the *GroEL* gene was performed based on the protocol described by Li et al. The first round utilized the outer primers Gro-1 (5′-AAGAAGGACGTGATAAC-3′) and Gro-2 (5′-ACTTCACGTAGCACC-3′), and the second round employed the inner primers TF1 (5′-ATATATCACAGTACTTTGCAAC-3′) and TR2 (5′-GTTCCTAACTTAGATGTATCAT-3′) for amplification of a 365 bp product [21]. The detailed PCR conditions (Tms and cycling parameters) are provided in the ( Supplementary Section Table S1). PCR was carried out in a 25 µL reaction volume, with 2 µL of template DNA in the first round and 1 µL of the PCR product in the nested round. PCR amplicons were resolved on 2% agarose gel alongside a 100 bp molecular ladder. Negative (no-template DNA) controls were included in each run. To avoid cross-contamination, only DNA extracted from human blood and previously confirmed as *O. tsutsugamushi* by sequencing was used as a positive control whenever a batch of samples failed to amplify completely. The PCR products of the expected size were subsequently purified and subjected to Sanger DNA sequencing in both the forward and reverse directions by the sequencing service provider, Barcode Biosciences, Bengaluru, India.

#### 2.3.2. Amplification of the *56-kDa* Type-Specific Antigen Gene

All the samples positive for the *GroEL* gene were further analyzed by nested PCR targeting the variable domains I-III (VDI–III) of the *56-kDa TSA* gene. The outer primers used were JG-OtF584 (5′-CAA TGT CTG CGT TGT CGT TGC-3′) and RTS9 (5′-ACAGAT GCA CTA TTA GGC AA-3′), followed by the nested primers F (5′-AGC GCTAGG TTT ATT AGC AT-3′) and RTS8 (5′-AGG ATT AGA GTG TGG TCCTT-3′), as per the method of Ruang-areerate et al. [14]. Amplification was confirmed by electrophoresis on 1% agarose gel. Positive PCR products were purified and subjected to Sanger sequencing. Details of the PCR reagents, thermal cycling conditions, and other technical parameters for both genes are provided in Supplementary Table S1.

#### 2.3.3. Sequence Editing, BLAST Analysis, and Submission

Forward and reverse sequence reads were manually reviewed using Chromas (version 2.6.6) [22] and assembled into consensus sequences using Windows Notepad (version 11.2504.62.0). The resulting sequences in FASTA format were aligned in MEGA 11 (Molecular Evolutionary Genetics Analysis version 11) [23] to verify the base-calling accuracy in the sequencing chromatograms. The edited nucleotide sequences in FASTA were searched using the BLASTn v2.17.0 tool in the NCBI GenBank database to determine genetic similarity and strain identity. A total of 33 *GroEL* and 11 *56-kDa* sequences corresponding to the IG-v clade were generated and submitted to GenBank (the accession numbers for *GroEL* and *56-kDa* are given in Tables S2 and S3).

#### 2.3.4. Phylogenetic Analysis

Phylogenetic trees were constructed for all 33 positive samples as well as separately for the 11 Gillam variants, using the maximum likelihood (ML) method in MEGA, version 11 [23]. The best-fit nucleotide substitution model for the dataset (n = 33 *GroEL* sequences) was determined using the model selection option. Referring to the lowest BIC (Bayesian Information Criterion) value, the T92 + G model was selected. Maximum likelihood (ML) phylogenetic trees were constructed using the Tamura 3-parameter (T3P) model with 500 bootstrap replicates, applying partial deletion and a 95% site coverage cutoff with a gamma-distributed rate. To further resolve the evolutionary relationships of the eleven IG-v sequences, a distance-based phylogenetic tree was constructed together with the Gilliam reference sequences. Model fit analysis yielded low BIC values, supporting the selected substitution model. For this dataset, the T3P parameters were applied with a uniform rate and complete deletion gap treatment, as the sequences were closely related and contained no gaps or deletions.

To complement the *GroEL*-based analysis, an ML tree of the *56-kDa* gene was generated using 11 representative sequences and prototype strains (Karp, Kato, Gilliam, and Ot-TJTN). In the comparative study, sequences representing prototype *O. tsutsugamushi* strains (Kato, Kuroki, Kawasaki, Ikeda, Hualien-1, Gilliam, Boryong, Hwasung, Karp, and TA716) were included. An ML tree was generated using the best-fit nucleotide substitution model (TN93 + G), determined according to the BIC, with the gap treatment set to partial deletion to account for informative gaps in the *56-kDa* sequences. The overall nucleotide diversity (π) and mean distance (d) between the IG-v (n = 11) vs. Gilliam-related strains were calculated.

#### 2.3.5. Similarity and Bootscan Analysis of *56-kDa* Sequences

To examine sequence similarity and detect possible genetic exchange events, SimPlot++ version 3.5.1 was used [24]. The three clades identified in the *56-kDa* sequences Gilliam-like, Karp-like, and Ot-TJTN-like were defined based on their phylogenetic clustering patterns. Among the 11 sequences analyzed, only the Karp-like and Ot-TJTN-like clades were independently assessed for similarity and recombination using bootscan detection. The single Gilliam-like study sequence (PV233813), which clustered closely with the Gillam prototype, was excluded from the Simplot++ because no recombination could be inferred for this clade. Bootscan was performed using the Kimura-2-parameter model, and similarity was assessed using the Hamming distance with a window size of 200 bp and a step size of 20 bp. Similarly, recombinant distance plots were generated using the IG-v study sequences as a query to detect potential recombination breakpoints.

## 3. Results

### 3.1. Molecular Detection and GroEL Gene Sequence Analysis

**Figure 1:**
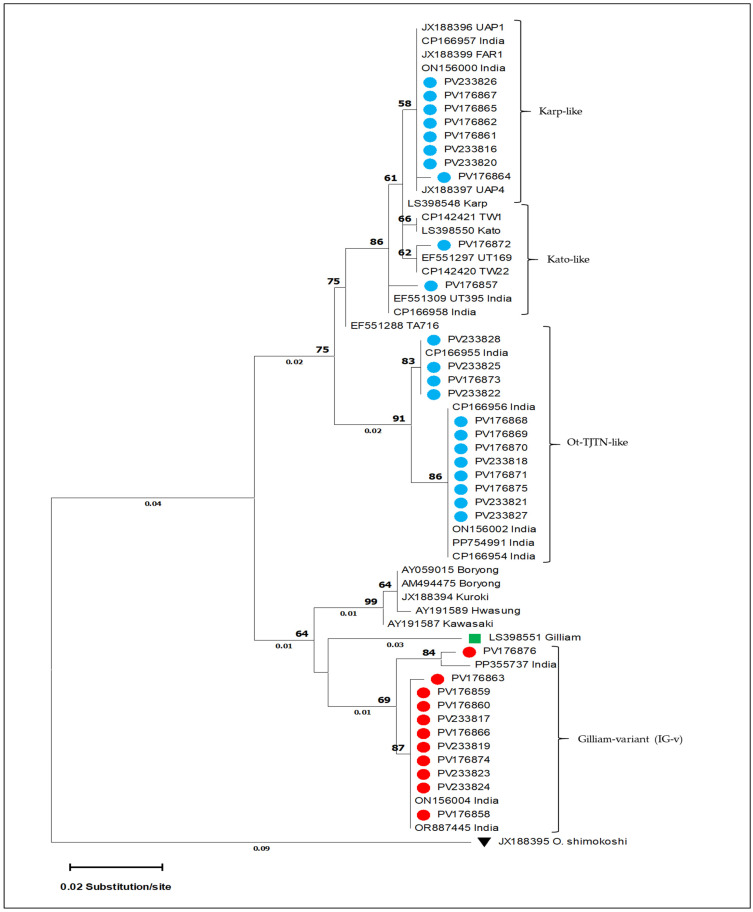
Maximum likelihood (ML) phylogenetic tree of the *groEL* gene constructed using the T3P model with 1000 bootstrap replicates. The tree resolves four major clades, viz., the Gilliam variant, Ot-TJTN-like, Kato-like, and Karp-like groups. Sequences generated in this study are highlighted in blue, with the IG-v cluster marked by a red bullet and the Gilliam reference sequence shown in green. Numbers above branches represent bootstrap support values, and numbers below indicate branch lengths. The tree is rooted using the reference outgroup (JX188965), indicated by an inverted triangle.

**Figure 2:**
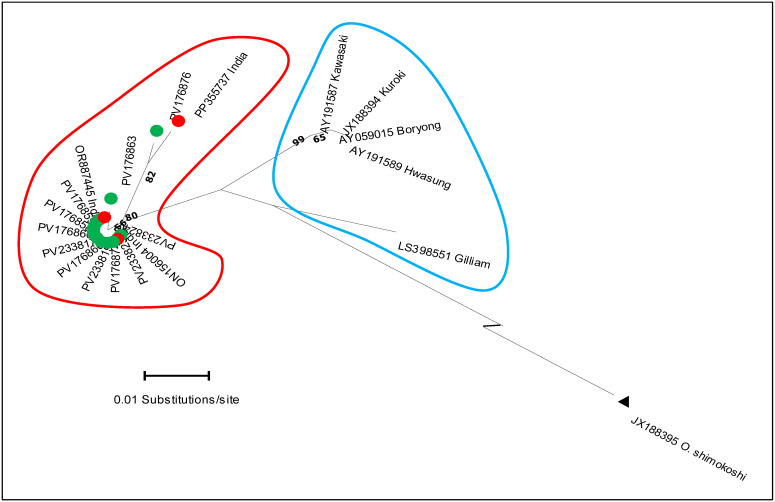
Maximum likelihood (ML) phylogenetic tree based on *GroEL* gene sequences, constructed using the radiation distance method. The tree shows two distinct clusters: the distinct Gv (red outline) and the Gilliam group reference strains (cyan blue). Only bootstrap support values greater than 50% are shown in the tree. Branches are labeled as follows: green circles represent Gilliam variant (IG-v) sequences, red represents Ot sequences from humans and rodents reported in previous studies from India, and the black triangle represents the outgroup.

A total of 105 clinical samples collected from the Theni (n = 51) and Dindigul (n = 54) districts were screened for *O. tsutsugamushi* using nested PCR, targeting the *GroEL* gene (Figure S1). Among these, 33 samples (31.4%) yielded high-quality sequences that were subjected to BLASTn analysis, revealing ≥96% nucleotide identity with previously reported *O. tsutsugamushi* strains in the NCBI database (Table S1). Phylogenetic analysis of the *GroEL*-365 bp fragment, using the ML method, resolved four major genotypic clusters: a Gilliam variant, Ot-TJTN-like, Kato-like, and Karp-like groups (Figure 1). For subsequent analyses with the *56-kDa* gene, only the 11 Gilliam variant sequences were included. Eleven of the thirty-three sequences (33.3%) formed a distinct monophyletic clade deviating from Gilliam and other known related strains (Figure 1 and Figure 2).

The estimation of the overall mean nucleotide diversity (π) was 0.02 (2%), and between the eleven IG-v/Gilliam, it was 0.039 (3.9%) (SE: 0.009). The nucleotide divergence between the IG-v and five Gilliam-related reference genotypes showed a divergence of >3%, indicating the presence of a distinct variant identified in this study (Table 1).

### 3.2. Sequence Analysis of the 56-kDa Gene

**Figure 3:**
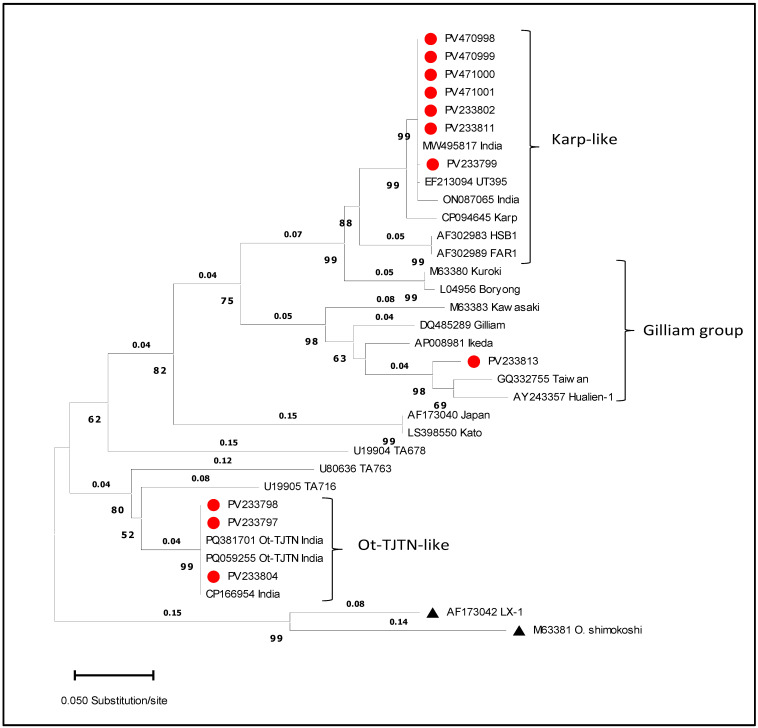
Maximum likelihood (ML) tree of the *56-kDa* gene constructed using the Tamura–Nei model (TN93 + G), as determined by BIC in MEGA 11 with 1000 bootstrap replicates. The tree shows three major clades: Karp-like, Ot-TJTN-like, and Gilliam-like. Numbers above branches represent bootstrap support values, and numbers below indicate branch lengths. Sequences from this study are indicated with red bullets. Two reference sequences, denoted by triangles, were used as outgroups to root the tree.

Eleven DNA samples, previously clustered within a distinct IG-v clade based on the *GroEL* gene, were further analyzed through nested PCR amplification targeting variable domains I–III (VDI-III) of the *56-kDa* gene (Table S1; Figure S2). A set of high-quality sequences was obtained from all eleven samples without background noise, enabling robust comparative phylogenetic analysis. Seven of the eleven identified as IG-v in the *GroEL* gene were clustered with the Karp-like strain in the *56-kDa* phylogenetic tree. These sequences exhibited 100% nucleotide similarity with the OT/India/0809aTw/2008 strain (MW495817) and 98.8% similarity with the isolates OT/AIIMS/4012/2021 (ON087065) from India and UT395 from Thailand (EF213094). Three of the remaining samples formed a well-supported cluster within the recently reported Ot-TJTN, displaying 100% homology with TJ49 (PQ381701) and the JJOtsu2C strain (PQ059255) from South India. Only one sample retained a Gilliam-like signature, aligning most closely with the TT0711a strain (GQ332755) from Taiwan (95.1%), the Hualien-1 strain (AY243357) from Taiwan (93.93%), and the CH01117 isolate from China (MT258819) (Figure 3). Based on the result of *56-kDa*, the 11 Gilliam variants were grouped (Table 2).

### 3.3. Similarity Plot and Recombination Analysis

**Figure 4:**
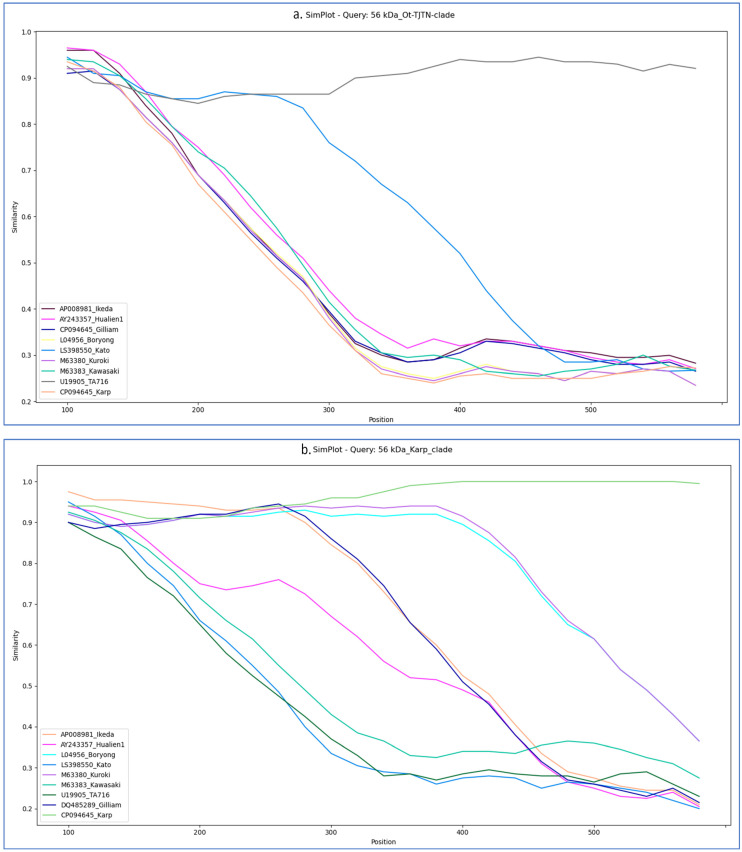
(**a**) Similarity plot (SimPlot) analysis of the *56-kDa* Ot-TJTN isolate against reference strains, showing the highest sequence similarity to TA716 and Kato. (**b**). Karp clade revealed the highest similarity to the Karp, Ikeda, Kuroki, and Boryong reference strains, supporting their classification within the Karp genotype. A stable flat curve across the genome suggests a consistent relationship to the reference throughout the region.

**Figure 5:**
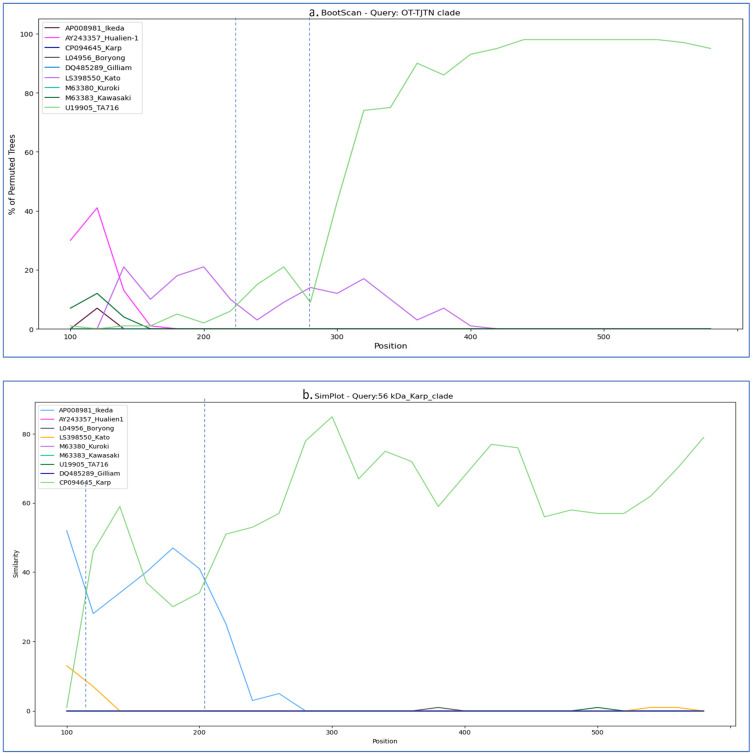
Bootscan analysis of *56-kDa* with the following parameters: Kimura 2-parameter (K2P) model, 200 bp window, 20 bp step size, and 500 bootstrap replicates. (**a**). Ot-TJTN isolate demonstrated evidence of sequence transfer involving TA716 and Kato strains; (**b**) Karp clade carries recombinant segments derived from both Karp and Ikeda lineages. Together, these patterns highlight the genetic exchange contributing to antigenic diversity in the *56-kDa* protein. The dashed vertical lines mark the recombination breakpoints within the *56-kDa* gene region.

SimPlot analysis of the Ot-TJTN isolate revealed maximum sequence similarity (≥90–95%) to the TA716 strain, followed by the Kato strain. Moderate similarity was observed with the Ikeda and Hualien-1 strains, whereas the other references displayed lower similarity profiles. Meanwhile, analysis of the Karp clade isolates showed the highest sequence similarity to the Karp, Ikeda, Kuroki, and Boryong reference strains across the *56-kDa* gene. All other strains exhibited significantly lower similarity, confirming the association of these isolates with Karp (Figure 4). The two clades in *56-kDa* were further investigated for potential genetic exchange breakpoints by using the bootscan analysis in Simplot++. The Ot-TJTN isolate displayed an event of recombination with the TA716 and Kato strains. Meanwhile, bootscan analysis revealed two crossover points within the *56-kDa* Karp-like clade, consistent with sequence mosaicism between the Karp and Ikeda strains (Figure 5).

## 4. Discussion

Changes in land use and host–vector movement enhance the zoonotic spread of scrub typhus [25], and this risk is compounded by the genetic diversity of *O. tsutsugamushi* reported across endemic regions [26]. Although the pathogen is primarily maintained through vertical transmission in mites, co-infections enable recombination between strains, generating genetically diverse variants with important epidemiological implications, such as altered virulence, diagnostic limitations due to antigenic mismatch, and challenges for vaccine development [27,28]. This diversity is shaped by gene-specific constraints. The *GroEL* gene, encoding a heat shock protein essential for survival, remains highly conserved and supports accurate phylogenetic classification [29,30], whereas the immunodominant *56-kDa* surface antigen gene, under strong immune selection, accumulates mutations and drives antigenic variability [29,31]. Together, these contrasting features make *GroEL* a stable marker for identification and phylogenetic characterization, while the *56-kDa* gene serves as an evolving marker of emerging genotypes and a critical target for region-specific vaccine strategies.

Based on this genetic foundation, our study focused on the molecular epidemiology of *O. tsutsugamushi* strains circulating among human scrub typhus cases in the Theni and Dindigul districts of Tamil Nadu, India. We identified a distinct clade in the Gilliam group and detected evidence of antigenic reassortment among circulating strains. These findings broaden the known genotypic diversity of *O. tsutsugamushi* in India and align with emerging reports of novel variants from other endemic regions in Asia [20,28,32]. Gilliam is one of the classical prototype strains, and it is known for its genetic variability across Southeast Asia. For instance, studies conducted in Thailand from 2004 to 2007 based on the *56-kDa* gene revealed several unique variants circulating in the country [14]. Similarly, a mosaic strain, TGv, has been reported in Taiwan, derived from the Gilliam, Ikeda, and Kato lineages [33]. Also, in India, a recent study from Karnataka documented the circulation of Japanese Gilliam (JG) and a Japanese Gilliam variant (JG-v) among scrub typhus cases, highlighting the ongoing diversification and geographic spread of Gilliam-related genotypes within the country [34].

Phylogenetic analysis based on the *GroEL* gene revealed four principal clusters. Among the four major clades, one monophyletic clade was distinct from classical Gilliam strains, which showed 100% identity with human clinical isolates from Theni and Thanjavur (GenBank: ON156004; OR887445) and 99.12% similarity with a rodent-derived isolate from Uttar Pradesh (PP355737), suggesting active zoonotic circulation with epidemiological significance. Furthermore, a mean nucleotide divergence of 3.9% between this IG-v clade and other known classical strains supports its preliminary classification as a genetically distinct variant. In addition, the observed nucleotide divergence of over >3% between IG-v and other known Gilliam-type variants suggests that this variant represents a first-detected molecular subtype of Gilliam circulating in the human and rodent populations. Interestingly, while *GroEL*-based analysis grouped all eleven isolates as Gilliam-related, the *56-kDa* gene phylogeny reassigned seven isolates to Karp-like, three to Ot-TJTN-like, and only one to Gilliam. The antigenically closely related Gilliam and Japanese Gilliam serotypes also differed in *56-kDa*-based genotyping (36). This discordance highlights how immune-driven variation at the *56-kDa* locus complicates lineage assignment if only immunodominant markers are used, reinforcing the need for multilocus typing in epidemiological studies [15].

SimPlot and bootscan analyses of the *56-kDa* gene sequences provided strong evidence of close genetic relationships among circulating strains. The results from the ML tree, similarity, and bootscan analyses were concordant for the Ot-TJTN clade, revealing a flat curve overlapping pattern and a recombination breakpoint between TA716 and Kato, indicative of genetic exchange. Analysis of the Karp-like sequences similarly showed high similarity to the Karp, Ikeda, and Kuroki reference strains, with evidence of recombination between the Karp and Ikeda genotypes. The involvement of Ikeda in the Gilliam variant has also been reported in Taiwan (34). These findings align with earlier studies showing that recombination, facilitated by co-infections within chigger mite populations, contributes substantially to the genetic heterogeneity of *O. tsutsugamushi*. Previous research has demonstrated high rates of homologous recombination, with multiple strains often co-infecting individual mites and enabling genetic exchange [4,35,36]. The ecological diversity of the Theni and Dindigul districts, including abundant populations of *L. deliense* and other trombiculid mite species (unpublished), likely favors such exchanges. Indeed, surveys in southern Tamil Nadu have documented several chigger mite species, with *L. deliense* identified as a predominant vector in these regions [37].

Identification of a distinct variant in the Gilliam clade, with a significant genetic divergence from classical Gilliam strains, reinforces its classification as a Gilliam variant of *O. tsutsugamushi* for the first time. Similar comparable levels of divergence have been associated previously with the emergence of the Kawasaki, Kuroki, and Boryong strains, indicating ongoing regional diversification [8,38]. Current serological assays, which rely primarily on classical antigens (Karp, Kato, Gilliam, and TA716), may fail to detect such divergent variants, leading to underdiagnosis in endemic regions. The strain-specific nature of immunity further complicates disease control, as previous infection does not ensure protection against emerging genotypes [39]. Genotyping based solely on the *GroEL* gene may mislead phylogenetic relationships; therefore, incorporating the more variable 56-kDa TSA gene improves accuracy in strain classification and epidemiological tracking [8]. The emergence of regional variants like IG-v reflects localized microevolution within the Gilliam group.

These preliminary findings represent the first detection of a Gilliam variant in this region and highlight the need for further investigation to determine whether it should be designated as a separate strain within the Gilliam group. Such confirmation will require analysis of larger fragment sizes or full-length sequences of the *GroEL*, *56-kDa*, and *47-kDa* genes, combined with additional recombination detection tools. This study also underscores the importance of updating diagnostic assays and strengthening molecular surveillance strategies to track the evolving genotypic diversity.

## 5. Conclusions

The high incidence of scrub typhus across Indian states urges the need for increased awareness of the pathogen diversity and the emergence of new strains for improved diagnosis and surveillance. Incorporating antigens from locally prevalent strains into serology-based diagnostic assays, instead of relying solely on standard reference strains, can reduce false negatives and improve case detection in endemic areas. This study highlights previously unrecognized dimensions of *O. tsutsugamushi* diversity in South India, notably the detection of a new Gilliam variant (IG-v) and evidence of ongoing antigenic reassortment. The discordance between phylogenetic signals from these two genetic markers underscores the complex evolutionary mechanisms shaping strain diversity in endemic regions. The emergence of such variants carries significant epidemiological implications, including genetic complexity, potential for drug resistance, challenges in accurate diagnosis, and barriers to developing protective vaccines.

## Figures and Tables

**Table 1:** Estimates of nucleotide diversity over sequence pairs between groups.

Genotype	Gilliam Variant (IG-v)	Hwasung	Kawasaki	Gilliam	Kuroki	Boryong
**Gilliam** **variant (IG-v)**	—					
**Hwasung**	0.039	—				
**Kawasaki**	0.032	0.006	—			
**Gilliam**	0.039	0.047	0.041	—		
**Kuroki**	0.035	0.003	0.003	0.044	—	
**Boryong**	0.035	0.003	0.003	0.044	0.001	—

**Table 2:** Genotypic classification of isolates based on the GroEl and *56-kDa* genes.

S. No.	*GroEL*		*56-kDa*	
	Accession Number	Genotype	Accession Number	Genotype
1.	PV176860	Gilliam-variant	PV233799	Karp-like
2.	PV176863	Gilliam-variant	PV233802	Karp-like
3.	PV233817	Gilliam-variant	PV470998	Karp-like
4.	PV233819	Gilliam-variant	PV470999	Karp-like
5.	PV176874	Gilliam-variant	PV233811	Karp-like
6.	PV233823	Gilliam-variant	PV471000	Karp-like
7.	PV233824	Gilliam-variant	PV471001	Karp-like
8.	PV176858	Gilliam-variant	PV233797	Ot-TJTN
9.	PV176859	Gilliam-variant	PV233798	Ot-TJTN
10.	PV176866	Gilliam-variant	PV233804	Ot-TJTN
11.	PV176876	Gilliam-variant	PV233813	Gilliam

## Data Availability

The original contributions presented in this study are included in this article; further inquiries can be directed to the corresponding authors.

## Supplementary Materials

The supporting information can be downloaded at https://www.mdpi.com/article/10.3390/microorganisms13122670/s1. Table S1. Details of primers and nested PCR conditions for amplifying *O. tsutsugamushi GroEL* and *56-kDa* genes; Table S2. Sample metadata and *GroEL* gene sequence similarity for *O. tsutsugamushi*; Table S3. Details of *56-kDa* gene sequences and BLAST analysis results corresponding to *GroEL*-positive samples; Figure S1. PCR amplification of the *GroEL* gene; and Figure S2. PCR amplification of the *56-kDa* gene.
