# Supplementary material for: A New Gilliam Genotypic Variant of Orientia tsutsugamushi in Human Scrub Typhus Cases from South India
Source: Microorganisms. 2025 Nov 24;13(12):2670. doi: 10.3390/microorganisms13122670 (PMC12734667; doi:10.3390/microorganisms13122670)

Table S1. Details of primers and nested PCR conditions for amplifying *O. tsutsugamushi* GroEL and 56-kDa genes

Gene	Primer Name	Sequence 5′ to 3′	Nested PCR	PCR Reagents	Thermal Cycler Conditions	Amplicon Size (bp)	Reference
**GroEL**	Gro-1<br>Gro-2	AAGAAGGACGTGATAAC<br>ACTTCACGTAGCACC	1st round	25 µL total volume: 2.5 µL 10× PCR buffer, 1 µL 25 mM $MgCl_2$, 1 µL 10 mM dNTP, 0.5 µL Taq polymerase (3U), 0.5 µL each primer, 2 µL template DNA, 17 µL nuclease-free water	95ºC 4 min; 37 cycles (94ºC 50 s, 45ºC 50 s, 72ºC 50 s); 72ºC 7 min; Hold at 4ºC	~364	Li et al.
	TF1<br>TR2	ATATATCACAGTACTTTGCAAC<br>GTTCCTAACTTAGATGTATCAT	2nd round	Same as 1st round, except 1 µL of first PCR product used as template DNA (All reagents from Genei, India)	95ºC 4 min; 37 cycles (94ºC 40 s, 56ºC 40 s, 72ºC 50 s); 72ºC 7 min; Hold at 4ºC		
**56-kDa TSA**	JG-OtF584<br>RTS9	CAATGTCTGCGTTGTCGTTGC<br>ACAGATGCACTATTAGGCAA	1st round	25 µL total volume: 5 µL 5× PCR buffer, 2 µL 25 mM $MgCl_2$, 1 µL 10 mM dNTP, 0.5 µL Taq polymerase (3U), 0.5 µL each primer, 2 µL template DNA, 13.5 µL nuclease-free water	94ºC 4 min; 39 cycles (94ºC 40 s, 51ºC 30 s, 72ºC 50 s); 72ºC 7 min; Hold at 4ºC	~700bp	Ruang-Areerate et al.
	F<br>RTS8	AGCGCTAGGTTTATTAGCAT<br>AGGATTAGAGTGTGGTCCTT	2nd round	Same as 1st round, except 1 µL of first PCR product used as template DNA (All reagents from Promega)	94ºC 4 min; 37 cycles (94ºC 40 s, 50ºC 30 s, 72ºC 50 s); 72ºC 7 min; Hold at 4ºC		

Table S2. Sample metadata and GroEL gene sequence similarity for *O. tsutsugamushi*

S. No.	Sample ID	Age/Gender	District	DNA Code	GenBank Acc. Nos. (Present Study)	Percentage Identity	Source	Country	Closest Acc. No.
1	500	23/M	Theni	TH 05	PV176858	100.00	Human	India	ON156004
						100.00	Human	India	OR887445
						98.11	Rodent	India	PP355737
						96.96	Human	Korea	AY191587
2	2540	6/F	Theni	TH 10	PV176859	100.00	Human	India	ON156004
						100.00	Human	India	OR887445
						96.98	Human	Korea	AM494475
						96.98	Human	Korea	AY191587
3	2465	7/M	Theni	TH 16	PV176860	100.00	Human	India	ON156004
						96.90	Human	Korea	AY059015
						96.90	Human	India	CP166955
						96.90	Human	Korea	AY191587
4	2525	6/F	Theni	TH 28	PV176863	99.73	Human	India	ON156004
						96.70	Human	Korea	AM494475
						96.70	Human	Korea	AY191587
5	2304	13/M	Theni	TH 45	PV233817	100.00	Human	India	ON156004
						98.11	Rodent	India	PP355737
						96.98	Human	Korea	AM494475
						96.98	Human	Korea	AY191587
6	49	45/F	Dindigul	DM 03	PV176866	100.00	Human	India	ON156004
						100.00	Human	India	OR887445
						96.70	Human	Korea	AM494475
						97.00	Human	Korea	AY191587

7	158	28/F	Dindigul	DM 14	PV233819	100.00	Human	India	ON156004
						100.00	Human	India	OR887445
						96.98	Human	Korea	AM494475
						96.98	Human	Korea	AY191587
8	1365	51/M	Dindigul	DM 30	PV176874	100.00	Human	India	ON156004
						100.00	Human	India	OR887445
						96.99	Human	Korea	AM494475
						96.99	Human	Korea	AY191587
9	1871	27/M	Dindigul	DM 33	PV176876	98.63	Human	India	ON156004
						99.12	Rodent	India	PP355737
						96.16	Human	Korea	AM494475
						96.16	Human	Korea	AY191587
10	1268	39/F	Dindigul	DM 43	PV233823	100.00	Human	India	ON156004
						100.00	Human	India	OR887445
						96.98	Human	Japan	JX188394
						96.98	Human	Korea	AY191587
						96.98	Human	Korea	AM494475
11	1696	55/M	Dindigul	DM 44	PV233824	100.00	Human	India	ON156004
						100.00	Human	India	OR887445
						96.98	Human	Japan	JX188394
						96.98	Human	Korea	AY191587
						96.98	Human	Korea	AM494475

Table S3. Details of 56-kDa gene sequences and BLAST analysis results corresponding to GroEL-positive samples

S. No.	DNA Code	GenBank Acc. Nos. (Present Study)	Percentage Identity	Source	Country	Closest Acc. No.
1	TH 05	PV233797	100	Human	India	PQ381701
			100	Human	India	PQ059255
			100	Human	India	CP166954
2	TH 10	PV233798	100	Human	India	PQ381701
			100	Human	India	PQ059255
			100	Human	India	CP166954
3	DM 3	PV233804	100	Human	India	PQ381701
			100	Human	India	PQ059255
			100	Human	India	CP166954
4	TH 16	PV233799	100	Human	India	MW495817
			98.84	Human	India	ON087065
			98.15	Human	Thailand	EF213094
5	TH 28	PV233802	100	Human	India	MW495817
			98.84	Human	India	ON087065
			98.15	Human	Thailand	EF213094
6	TH 45	PV470998	100	Human	India	MW495817
			98.84	Human	India	ON087065
			98.15	Human	Thailand	EF213094
7	DM 43	PV471000	100	Human	India	MW495817
			98.84	Human	India	ON087065
			98.15	Human	Thailand	EF213094

8	DM 44	PV471001	100	Human	India	MW495817
			98.84	Human	India	ON087065
			98.15	Human	Thailand	EF213094
9	DM 14	PV470999	100	Human	India	MW495817
			98.84	Human	India	ON087065
			98.15	Human	Thailand	EF213094
10	DM 30	PV233811	100	Human	India	MW495817
			98.84	Human	India	ON087065
			98.15	Human	Thailand	EF213094
11	DM 33	PV233813	95.14	Human	Taiwan	GQ332755
			93.93	Human	Taiwan	AY243357
			93.93	Human	China	MT258819
			93.93	Human	Taiwan	GQ332754
			92.03	Human	Japan	AP008981

Figure S1. PCR amplification of the *O. tsutsugamushi* GroEL gene. Sample lanes as labeled with DNA code, DL:DNA Ladder, NC: negative control. The ~364 bp PCR product is indicated by an arrow.

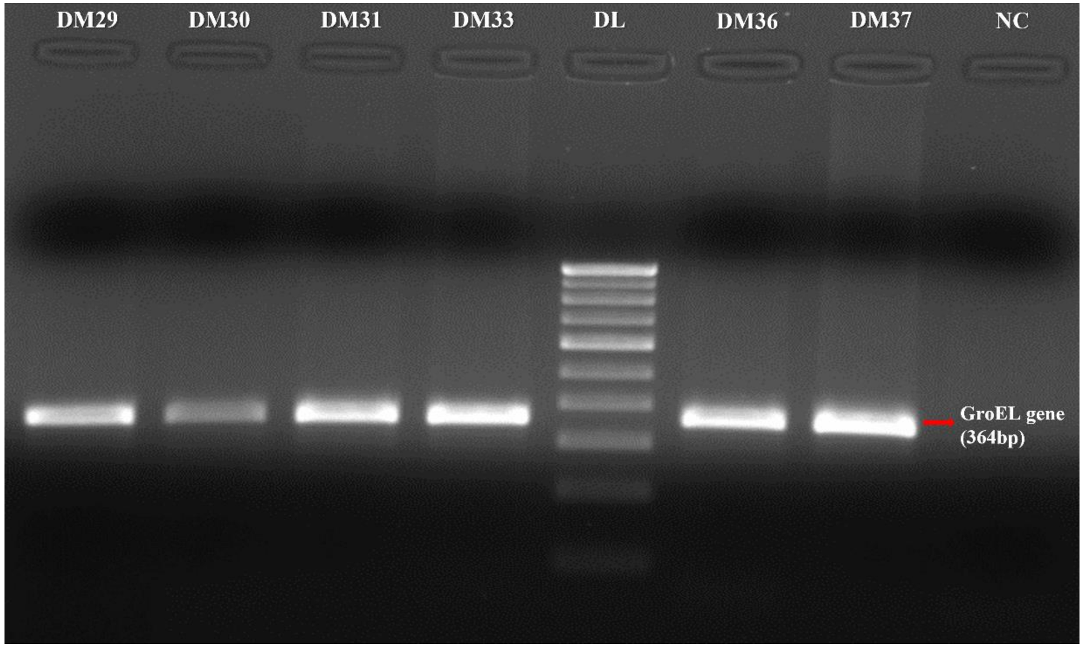

Figure S2. PCR amplification of the *O. tsutsugamushi* 56-kDa gene. Sample codes are indicated above each lane. DL: DNA ladder (0.1–3 kb); BL: blank; NC: negative control. The ~700 bp PCR product is indicated by an arrow

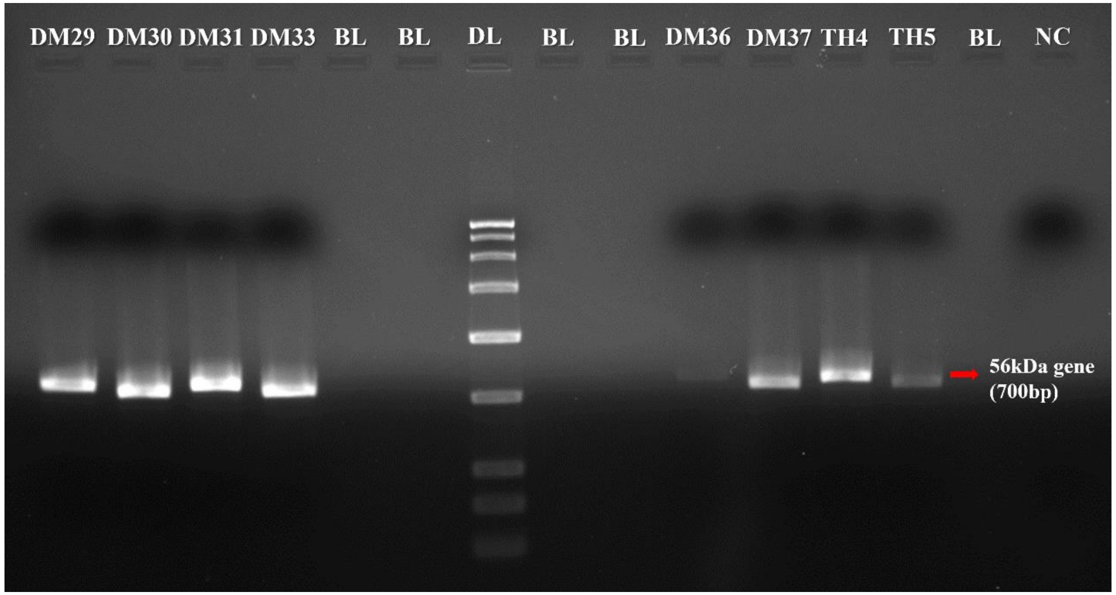

Supplement: Supplementary file 1 [file microorganisms-13-02670-s001.zip › microorganisms-3907746-supplementary.pdf]
